# Dependent Personality Disorder and Intimate Partner Violence: the “Perfect Marriage”

**DOI:** 10.1192/j.eurpsy.2023.2057

**Published:** 2023-07-19

**Authors:** B. R. Afonso, R. S. Carvalho, F. M. Silva

**Affiliations:** Psiquiatria, Hospital de Magalhães Lemos, Porto, Portugal

## Abstract

**Introduction:**

Intimate partner violence (IPV) is broadly defined as physical, sexual, or psychological harm inflicted by a current or former romantic partner or spouse. Unfortunately, even nowadays, the prevalence rates of IPV victimization are still very high, with over one third of women reporting any contact sexual violence, physical violence, and/or stalking and nearly half having psychological aggression in their lifetime. Amongst a complex network of risk factors for IPV, Personality disorders (PD) are one of the most researched. Defined as enduring patterns of inner experiences and behaviors, PD play a significant role in IPV, causing perpetrators to recidivate and victims of IPV to stay in violent relationships.

**Objectives:**

A case based approach is used to illustrate the association between Dependent Personality Disorder and Intimate Partner Violence

**Methods:**

Case Report and Brief Literature review

**Results:**

Case: We present a case of a 65-year-old woman, long term followed by psychiatry for anxiety and depression symptoms, built upon a personality with dependent traits. The patient has been victim of intimate partner violence since her marriage, more than 40 years ago, generating significant psychopathology, functional impairment and several suicidal attempts over the years. Nonetheless, the patient feels emotionally attached and has pity for her husband. Despite this abuse had been already reported, the patient has been unable to act on the complaint, perpetuating this situatuon over time. The insight is totally preserved since the victim has full conscience of the causality between perpetrator cumulative abuse and her psychological and physical suffering. This case supports research in this area which had found that individuals with high levels of dependent PD traits tend to have higher ratings of relationship satisfaction and see their relationship in a more positive light, causing them to downplay the IPV they experience.

**Conclusions:**

The prevalence of IPV poses a serious public health concern, particularly given the increased risk of physical and mental health problems that have been linked to IPV, such as chronic pain, depression, post traumatic stress disorder, substance use, and suicidal ideation, as well as a host of other negative outcomes. Individuals with high levels of dependent PD traits are prone to victimization, clinicians should be alert.

**Disclosure of Interest:**

None Declared

